# 

**Published:** 1924-04

**Authors:** 


### PUBLIC HEALTH IN PARLIAMENT.

The following have been selected, as being of general interest, from recent answers to Parliamentary questions.

### HOUSING.

Progress.—Up to February 1, 22,600 houses had been commenced under the Housing, etc., Act, 1923, and 4,680 had been completed. There were also 4,634 houses under construction on this date under the previous housing scheme. The corresponding annual rate of construction is difficult to estimate, but may be put at from 36,000 to 40,000 houses a year. Up to the same date approval has been given by the Ministry of Health to schemes proposed by local authorities for 95,715 houses, of which 33,994 are to be erected by local authorities and 61,721 by private enterprise. In respect of the houses to be erected by the local authorities themselves, contracts have been entered into covering 18,687 houses. As regards the houses to be erected by private enterprise, certificates have been issued on the approval of plans for 36,543 houses. In a Circular issued in August last local authorities were informed that it was not proposed to prescribe a maximum density. The opinion was expressed, however, that a density of approximately twelve houses to the acre represented a desirable standard, and that as a general rule a local authority should not approve the building of more than twenty houses on any one acre. It is not my intention to modify this policy.

### NATIONAL HEALTH INSURANCE.

Consolidation Bill.—A Bill to consolidate the National Health Insurance Acts was introduced in another place on February 28, was read a second time on March 4 and is now before the Joint Committee on Consolidation Bills.

Appointment of Royal Commission.—The Government are considering the setting up of a Royal Commission as soon as arrangements can conveniently be made to enquire into and make recommendations as to the whole system of National Health Insurance.

Social Insurance.—I do not think it would be desirable to extend the terms of reference of the proposed Royal Commission in the manner suggested, to cover the whole field of Social Insurance. There is ample material for investigation within the limits proposed. The better co-ordination of the various social services is a separate matter, which is receiving consideration.

### VOLUNTARY HOSPITALS.

The Financial Position.—The position of the voluntary hospitals, though it is still difficult, has greatly improved since the report of Lord Cave's Committee. The proposal contained in the second part of the question, to introduce a comprehensive health system on a national basis, is one with which the Minister of Health has some sympathy, but it raises far reaching issues, both medical and financial, and he is not at present prepared to give any undertaking as to it.

### TUBERCULOSIS.

Spahlinger Treatment.—I am anxious to arrange for a scientific investigation of this method of treatment in this country, and have personally given Mr. Spahlinger assurances to that effect.

### DIPHTHERIA.

Preventive Treatment.—In a reply to a question whether the Minister's attention has been drawn to the remarkable results obtained by the Department of Health in New York in immunising children against diphtheria, Mr. Wheatley said: This form of preventive treatment was the subject of a special medical report by my Department in 1921, and in July, 1922, a memorandum was issued to local authorities drawing attention to the practical advantages of the method. The question of offering the treatment to parents is for the decision of local authorities or the managing bodies of institutions, but my Department has offered expert advice and assistance in the application of the treatment by those who desire to adopt it.

Schick's Reaction Test.—I am not aware that any permission on my part is required in this matter. Local Authorities have been advised by my Department that the consent of the parents or guardians should be obtained before the test is applied to children.

### RECENT APPOINTMENTS.

### Medical Officers, Chaplains, &c.

Ashley, T. E., M.R.C.S., L.R.C.P.; M.O.H. Portishead.

Bloomfield, A., M.D., F.R.C.S.; Assistant Surgeon, South London Hospital.

Borthwick, G. A., M.D., Ch.B.; M.O., Port of Plymouth.

Bull, W. E. H., M.C., M.R.C.S., L.R.C.P.; M.O.H., Hunstanton.

Downing, Ethel, M.R.C.S., L.R.C.P.; House Surgeon, New Sussex Hospital for Women and Children, Brighton.

Forrester, A. T. W., M.D., M.R.C.S., L.R.C.P., Deputy Medical Superintendent, Leicester and Rutland Mental Hospital; Medical Superintendent, Warwickshire and Coventry Joint Mental Hospital.

Jennings, Henry Cecil, M.B., Assistant M.O. and Chief Sanitary Inspector, Leeds. Temporary M.O.H. and Schools M.O., Holland County Council (£1,100 and travelling expenses).

Jones, David Thomas Rocyn, C.B.E., M.D., D.Ph., M.O.K. for Monmouthshire; Knight of Grace of the Order of St. John of Jerusalem.

Macdonald, J. B., M.A., M.B., Ch.B.; Resident M.O., East Poorhouse Hospital, Dundee; Senior Resident M.O. Poorhouse Hospital, Dundee.

Redman, C. E., M.R.C.S., L.R.C.P.; Senior Resident M.O., Winsley Sanatorium.

Simpson, J. U. A., M.D., D.P.H.; Deputy M.O.H., Torquay.

Smith, Hoyland, M.R.C.S., L.R.C.P.; Police Surgeon, Leeds Corporation (£1,000).

Watson, F. H., M.B., Ch.B.; M.O., Maternity Hospital and Clinics, Portsmouth.

Williams, Enid Ann, L.R.C.P., L.R.C.S.; Assistant Resident M.O., Wolverhampton Poor Law Institution.

### Public Health and Hospital Appointments.

Duesbury, Muriel, Assistant Matron, Royal Southern Hospital, Liverpool; Matron, Doncaster Royal Infirmary.

Eldridge, Miss, C.S.M.M.G.; Masseuse, New Sussex Hospital for Women and Children, Brighton.

Eyton, Emily Gertrude, School Nurse, Maidstone.

Francis, Ernest, Chief Clerk, Chester Royal Infirmary; assistant to superintendent and secretary, Preston Royal Infirmary.

Gorniot, T.; Chief Sanitary Inspector, Marylebone.

Hester, Georgina, Matron, Hounslow Hospital.

Matthews, Beatrice; Matron, Didworthy Sanatorium, South Brent.

Nettell, Nelly; Matron, Carr House Infectious Hospital, Doncaster.

Payne, Mary Sutherland; County Health Superintendent, Gloucestershire.

Pearson, Grace D. B., Sister, West End Hospital for Nervous Diseases; Matron, Lady Chichester Hospital for Early Breakdown, Brighton.

Perry, Gertrude Annie; County Health Superintendent, Gloucestershire.

Prydderch, Mary, Health Visitor and School Nurse, Cardiganshire.

Pullar, Isabella M.; Health Visitor, West Suffolk.

Smith, I.; Matron, Grindon Convalescent Home.

Stickland, E. E.; Matron, Urban District Council Isolation Hospital, Sevenoaks.

Thaw, Whilloamina; Superintendent, Queen Victoria Jubilee Nurses Institute, Greenock.

Thomson, M. B., Assistant Matron Oxford Eye Hospital; Matron, United Services Fund Holiday Home for Tuberculous ex-Service Men.

Turner, Clara E.; Health Visitor, Hertfordshire County Council.

Walton, Margaret; Tuberculosis Health Visitor, Salford.

White, Dorothy, C.M.B., Health Visitor, Dorset County Nursing Association.

Williams, F. M.; Superintendent Nurse, Solihull Union Infirmary.

### ANSWERS TO CORRESPONDENTS.

Regular Reader.—Medical men with a recognised Canadian qualification can practice in England upon registering their qualification here.

X.—The Secretary of the Royal Medical Benefit Fund is Mr. G. Bethel, 11, Chandos-street, W. 1.

A.P.—Sir Humphrey Rolleston is the President of the Royal College of Physicians and Sir John Bland Sutton President of the Royal College of Surgeons.

W.B.L.—The book referred to was "Womanhood and Health," by Christine N. Menell (Mills and Boon. 5s. net.).

Charles Gates (Newcastle).—Gamgee tissue is a speciality of Messrs. Robinson & Sons, Ltd., the surgical dressings manufacturers of Wheatbridge Mills, Chesterfield.

B. B.—For laundry requirements you would do well to apply to Messrs. W. R. Hatton & Sons, Ltd., of Wormwood Scrubbs, W. 10. This firm manufactures starch, blue and laundry equipment generally.

Sister B.—(1) Yes, certainly. (2) You could obtain excellent waterproof bed sheeting from the Provincial Waterproof Co., Ltd., of Provincial Works, Strangways, Manchester.

Distemper.—We recommend you to try Messrs. Carson & Sons, the well-known makers of distemper, varnishes and enamels.

G. Singleton.—The National Conference on the Prevention of Destitution was held, under the presidency of the then Bishop of Oxford, on June 11, 12, 13 and 14, 1912. A full report was afterwards published by Messrs. P. S. King & Son.

### BUBBLING FOUNTAINS.

A form of drinking fountain in common use in America, particularly among schools, might well be adopted more freely in this country. The principle is that a fine jet of water rises from the fountain, and over this the child may place its lips and drink without touching the fixture or allowing mucus or saliva to contaminate the fountain for the next comer. Admittedly all fountain models so constructed are not proof against disease-spreading, for in some, unless the child is carefully instructed in their use, contamination may occur as freely as with any common cup. If, however, the water spouts out more or less horizontally from a covered opening, the lips cannot possibly touch the opening and, given a pure and proper water supply, risk of infection is practically nil.

### SOME "GHASTLY REVELATIONS."

In an address to the students of St. Mungo's College, Glasgow, Sir Leslie Mackenzie said that if they read the report by the Ministry of National Service on the physical examination of men of military age from November 1, 1917, to October 31, 1918, they would find some ghastly revelations. The number of men examined or re-examined was round about 2,500,000. The medical examinations showed that of every nine men of military age in Great Britain on an average three were perfectly fit and healthy, two were upon a definitely infirm plane of health and strength, three were incapable of undergoing more than a very moderate degree of physical exertion, and could almost, in view of their age, be described with justice as physical wrecks. The remaining man was a chronic invalid with a precarious hold on life. That was the greatest single investigation ever made in this country. With every qualification the result was staggering.

### Managerial Notices.

*All letters on Business matters must be addressed to the Manager, "The Hospital and Health Review," 28 and 29 Southampton Street, London, W.C. 2.*

RATES FOR SUBSCRIPTION (payable in advance).

| | | | |
|---|---|---|---|
| Three Months (including Postage) | | 1s. | 9d. |
| Six Months | do. | 3s. | 6d. |
| Twelve Months | do. | 7s. | 0d. |

**It is especially requested that remittances be made by Cheque or Postal Order and not Stamps.**

Subscriptions, which may commence at any time, are payable in advance. Cheques and Post-Office Orders should be crossed Westminster Bank, Covent Garden Branch, and made payable to the Manager of THE HOSPITAL AND HEALTH REVIEW.

***Advertisements.*** Scale of charges and particulars of special positions may be obtained on application to the Manager.

### *SUBSCRIPTION FORM.*

*Please send me* THE HOSPITAL AND HEALTH REVIEW *for*............ *months, commencing with your issue of* ..............., *for which please find enclosed* .......

*Name* ..............................

*Address* ..............................

